# Implementing an Acute Frailty Service in the Emergency Department: A Mixed‐Methods Service Evaluation of Feasibility, Patient Outcomes and Experience

**DOI:** 10.1111/jep.70432

**Published:** 2026-03-30

**Authors:** Yuhan Zhang, Victoria Green, Alex Montagu

**Affiliations:** ^1^ Faculty of Health and Life Sciences Oxford Brookes University Oxford UK; ^2^ Medicine, Rehabilitation and Cardiology Department Oxford University Hospitals NHS Foundation Trust Oxford UK; ^3^ Surgical Liaison Service Royal Berkshire Hospital NHS Foundation Trust Oxford UK

**Keywords:** emergency department, frailty, geriatric emergency medicine, implementation, patient experience, service evaluation

## Abstract

**Background:**

Older adults living with frailty present frequently to emergency departments (EDs), yet standard care is poorly adapted to their complex needs. National policy in England recommends Comprehensive Geriatric Assessment (CGA) and frailty‐targeted pathways, but evidence on their impact in EDs is limited.

**Methods:**

We conducted a single‐centre mixed‐methods service evaluation using an observational comparative design to evaluate a new frailty service in a large urban ED (September 2023–March 2024), based on routinely collected data (Commissioning for Quality and Innovation (CQUIN) dataset) and a patient experience survey.

**Results:**

Among 5,717 eligible ED presentations (clinical frailty scale (CFS) ≥ 6), 464 (8.1%) received the acute frailty service (AFS) review and 5,253 (91.9%) usual care. AFS patients were older (mean 85.9 (SD 7.7) vs 81.0 (SD 12.3) years) and more often female (64.2% vs. 55.3%). Overall LOS was shorter for AFS patients (49.1 ± 48.5 h ≈ 2.0 days vs 106.4 ± 95.5 h ≈ 4.4 days; *p* < 0.001), driven primarily by admitted patients. Admission avoidance was achieved in 23% of AFS cases. Odds of hospital admission were lower (OR 0.14, 95% CI 0.087–0.231), while 30‐day reattendance did not differ significantly (OR 0.84, 95% CI 0.51–1.36). No in‐hospital deaths occurred among AFS‐reviewed patients. Patient feedback (*n* = 24) showed high satisfaction with dignity, communication, and overall care. A simple cost‐offset estimate suggested two admissions avoided per clinician shift, approximating local bed‐day cost savings of £1,000 per day.

**Conclusions:**

Implementing a dedicated frailty service in the ED is feasible and may improve clinical outcomes and patient experience. Embedding specialist geriatric input early in the emergency pathway supports safe, efficient, and person‐centred care for older adults. These findings offer transferable insights for acute and emergency care systems seeking to strengthen frailty management at the front door.

**Statement of Significance:**

This study provides real‐world evidence on implementing an acute frailty service within the emergency department and describes its reach, integration, and associated early outcomes for older adults living with frailty. The findings contribute to international efforts to optimise frailty management at the front door of acute hospitals, offering practical insights for integrated emergency and geriatric care.

## 1 Background

Global data paints a stark picture: The number of people over 65 is projected to surge by 48% in Europe and North America by 2050 [1]. This demographic shift is not without consequences. International evidence indicates that older adults already account for a significant 25% of emergency department (ED) attendances [2]. Patients with frailty are at a heightened risk of hospital admission, disability, and even death [3]. These demographic and clinical pressures have prompted increasing policy focus on “front door” frailty models that embed specialist geriatric expertise early within emergency care pathways. Older adults living with frailty often present with complex needs that require early recognition and coordinated, multidisciplinary responses to optimise outcomes. Despite growing policy emphasis on “front door frailty” models [4], implementation within ED settings remains inconsistent and under‐evaluated.

Several international models have sought to address this gap. An Italian frailty network [5] advocated and utilised multidimensional evaluation to approach frailty in the ED. In their model of care, they identified older adults with frailty who were incongruous with hospitalisation and who were predicted to have a complex discharge process. In this cohort of patients, their cognitive‐functional problems were assessed and addressed by formulating a continuity of care pathway. The care pathway might include admission to a cognitive‐functional unit, referral to general practitioners (GPs) or palliative care, or discharge home with care or back to care homes with specialist home visits. The network demonstrated improved identification of frail older adults, more appropriate admission decisions, and enhanced continuity of care between hospital and community settings, reducing unnecessary hospitalisations while maintaining patient safety [4]. These findings informed the development of our acute frailty service, particularly the emphasis on multi‐disciplinary assessment and integration with community pathways to support admission avoidance.

Research by Bogucki et al. [6] suggests that follow‐up care for patients admitted to the ED after a fall, including paramedic or nurse visits at home, is associated with a significant reduction in all‐cause ED utilisation for up to 90 days, through enhancing the health and safety of older adults, preserving their independence, and improving their quality of life. This is further supported by another study, which found that community‐based comprehensive geriatric assessment (CGA) improved utilisation of primary care, continuity of care and hospital reattendance rates [7]. These findings underscore the importance of integrating community services into ED frailty pathways—a core design feature of our acute frailty service. Rather than viewing community care as a post‐discharge afterthought, our service operationalises these evidence‐based interventions from the point of ED arrival by establishing direct referral pathways to hospital‐at‐home teams, gerontology clinics, care home support services, or fall prevention teams. This enables the acute frailty service (AFS) team to initiate community support immediately, facilitating early safe discharge while ensuring continued multidisciplinary follow‐up for older adults living with frailty.

The Commissioning for Quality and Innovation (CQUIN) 2023/24 guidelines from NHS England [8] state that all patients aged 65 and above who present to the ED should undergo a clinical frailty scale (CFS) assessment, and that a CGA should be initiated for individuals with a CFS score of 6 or above. Initiation of CGA involves five domains: medical, functional, social, cognitive, psychological, and environmental [9]. Since this assessment involves multiple professionals and is an iterative process, NHS England and NHS Improvement [10] emphasise the need for an AFS that connects health systems and healthcare professionals across hospitals and community services. This study, therefore, sought to evaluate the real‐world implementation of an AFS in the ED, integrating geriatric expertise into the early phase of emergency care, and to examine its feasibility, clinical outcomes, and patient experience.

### 1.1 Developing an Acute Frailty Service From ED to Community

Following CQUIN 05 2023/2024 [8], an AFS was developed in the ED of an acute hospital to deliver CQUIN 05 and reduce unnecessary hospital admissions. The long‐term strategy is to have 40 to 50 virtual wards for every 100,000 people in 2023/2024 [11]. Frailty pathways were developed as a quality‐improvement project to enhance the identification and care of older adults with frailty. Existing local services include the Ambulatory Outreach Team (AOT), which provides both a virtual ward telephone service and Hospital at Home (H@H) teams that deliver hospital‐level treatment and care directly to patients' homes. A geriatrician‐led Urgent Community Response (UCR) service provides clinical oversight and remote advice. These resources offer hospital‐level care in patients' own homes, thereby providing alternative options to hospital admission.

### 1.2 Effectiveness of CGA in ED

Older people with frailty have worse outcomes in the ED than other patient groups. Therefore, the delivery of holistic ED‐based interventions targeting medical, functional, psychological, and social aspects [10] is required to provide better care and to meet the needs of older adults living with frailty who present to the ED [12]. However, providing AFS in the ED is not without complex issues. A systematic review of frailty prevalence and outcomes conducted by Boucher et al. [13] recommended differentiating varying degrees of frailty when formulating guidelines and policies. However, the details of implementing this differentiation remain unclear. Various studies have shown the benefit of CGA in preventing institutionalisation [14] and improving quality of life [12]. CGA has been widely adopted across multiple settings, including acute hospitals [12], communities [15, 16], hospitals at home [17], and among oncology patients [18]. However, the evidence for CGA in ED remains insufficient [19, 20]. Therefore, evidence on ED‐based frailty services remains sparse. This study contributes unique real‐world data on feasibility, outcomes, and patient experience, addressing a critical gap in the literature.

### 1.3 The Acute Frailty Service Design

The AFS comprises a senior nurse and a Specialist Registrar in Geriatric Medicine, with consultant advice available as needed. In conjunction with the therapist team, the frailty intervention team (FIT), the AFS team routinely screens the ED patients' list and identifies patients (age 65+) who are moderately frail (CFS ≥ 6). The ED doctors and nurses also highlight relevant patients for the team to review. The criteria for AFS review are outlined in Table 1.

**Table 1:** Acute frailty service review criteria.

Criteria for selecting eligible patients for the frailty service
1. Patients age ≥ 65 years with a CFS ≥ 6
2. Patients with CFS < 6, who may benefit from CGA
3. Patients who present with a Frailty Syndrome (falls, immobility, delirium, incontinence or medication side effects)
4. Patients who are suitable for admission avoidance with involvement of ambulatory or community services

**Figure 1:**
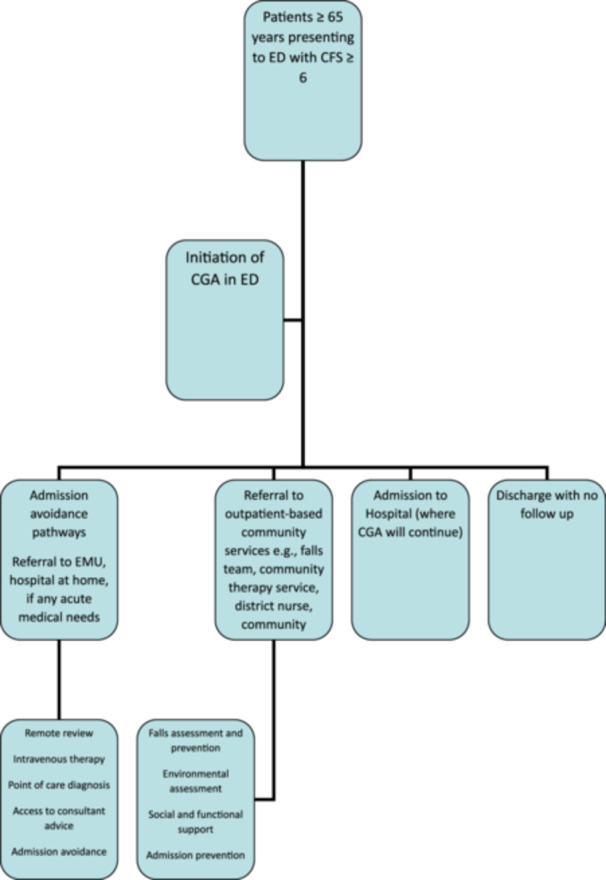
Flow chart of the frailty pathway.

**Figure 2:**
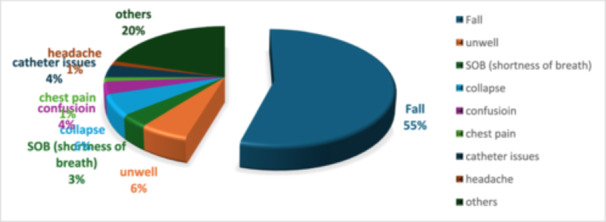
Patients' main presentations seen by AFS in ED.

The clinical pathway (Figure 1) includes initiating CGA for eligible patients. If an acute medical need can be met with H@H visits or virtual ward telephone review services, an appropriate referral will be made to support early and safe discharge. The service also utilises community resources, such as the falls team, the care home support service, the community palliative team, the gerontology clinic, and emergency multidisciplinary units. Patients' main presentations to the ED that AFS sees are outlined in the pie chart (Figure 2).

## 2 Aim

This mixed‐methods service evaluation aimed to assess the feasibility of implementing an AFS within a busy ED and to examine its early clinical outcomes and patient experience. Feasibility was evaluated across four domains: reach (the proportion of eligible frail older adults reviewed by the service); integration (whether the service could be embedded without prolonging ED length of stay for discharged patients); admission avoidance capability (the successful utilisation of community pathways to prevent hospital admissions); and acceptability (patient satisfaction with the care received). Secondary aims were to examine early clinical and service outcomes, including hospital length of stay, hospital admission rates, 30‐day ED reattendance, inpatient mortality, and to provide a preliminary estimate of cost‐offset based on admissions avoided.

## 3 Method

### 3.1 Design and Setting

This was a single‐centre mixed‐methods service evaluation conducted in a large urban ED between September 2023 and March 2024. Routine clinical data were collected as part of service delivery and CQUIN monitoring and subsequently extracted retrospectively for evaluation. The evaluation followed NHS Health Research Authority guidance for service evaluations and was not designed to establish causality. This service evaluation is reported in accordance with the STROBE checklist (Appendix 1).

### 3.2 Sample Size and Selection

A total of 464 patient records were included. The sample consisted of all patients meeting the inclusion criteria during the evaluation period. The sample size was determined pragmatically, based on the number of eligible patients available.

### 3.3 Intervention

The intervention is described below, following the Template for Intervention Description and Replication (TIDieR) framework [21].

**Brief name of the intervention:** AFS – ED‐based CGA and admission avoidance pathway.

**Why:** The service was developed to deliver CQUIN 05 requirements, improve identification of older adults with frailty, initiate early CGA, reduce unnecessary hospital admissions, and facilitate early community support through integrated discharge pathways.

**What (materials):** The team uses the ED patient electronic system and CFS to identify potentially eligible patients. Clinical assessment follows CGA domains using locally adapted proformas covering medical review, medication reconciliation, functional assessment, cognitive screening, social circumstances, and advanced care planning discussions. Referral pathways to community services are standardised with electronic referral forms and criteria checklists (e.g., two serious falls a year warrant a falls prevention team referral).

**What (procedures):** The AFS team conducts daily reviews of ED attendees, identifies eligible patients, performs initial CGA, develops a management plan in conjunction with the ED team, and initiates referrals to community services where appropriate. The team liaises with the FIT team for therapy assessments and with the ED medical team for acute care coordination.

**Who provided:** One senior nurse (Band 8a) and one full‐time Specialist Registrar in geriatric medicine (ST5 + ), with consultant geriatrician advice available via telephone or in‐person review as needed.

**How:** Face‐to‐face assessment in the ED cubicles. Telephone liaison with community teams, GPs, care homes, and families.

**Where:** Large urban ED in a teaching hospital. Assessments conducted in ED clinical areas; community follow‐up delivered in patients' own homes or care homes.

### 3.4 When and How Much

- **Service hours:** Monday–Friday, 9 a.m.–5 p.m. (extended to 8 pm for FIT).
- **Duration and frequency of intervention:** Single episode of CGA initiation in ED, with follow‐up delivered by community teams (duration varies by pathway, e.g., H@H visits typically 3–7 days).

**Tailoring:** Intervention was tailored based on:

- Clinical presentation (falls, delirium, immobility, etc.).
- Acuity (NEWS2 score).
- Baseline frailty level (CFS score).
- Social circumstances and existing care package.
- Patient and family goals of care.
- Availability of community services.

**Modifications:** None during evaluation period. Service operated as designed from September 2023.

**How well (fidelity):** No formal fidelity assessment was conducted. Service delivery was determined by the clinician based on eligibility criteria. A proportion of eligible patients were not seen due to service capacity and operating hours.

### 3.5 Data Collection

Data collection was anonymised and evaluated outcomes, including patient age, CFS score, length of stay, therapy review, geriatrician review, admission/discharge, and reattendance (to ED) in 30 days. Where the AFS or FIT were not involved, we reviewed clinical notes to identify equivalent assessments conducted by other professionals—for example, mobility assessments by medical staff or ward nurses (as a proxy for therapy review), and medication reconciliation by pharmacists or prescribing decisions by ED doctors (as a proxy for geriatrician review). These record reviews were conducted by AFS clinicians, and any ambiguous cases or uncertainties were resolved through discussion and double‐checking between reviewers. Patients not reviewed by AFS were classified as receiving usual care, defined as standard ED assessment and management without structured AFS review.

Hospital informatics support was requested to complement the manually collected data. The informatics team extracted reattendance data from hospital records using a standardised report, identifying any unplanned return to the ED within 30 days of the index attendance. Reattendance was defined as any emergency presentation for any cause, not limited to the same presenting complaint. The informatics team was blinded to whether patients had received AFS intervention, as they extracted data solely on the basis of patient identifiers and date parameters, without access to clinical involvement data.

Admission avoidance was defined as discharged patients who would have been admitted if not for AFS intervention. Admission avoidance numbers were collected routinely by the AFS clinicians at the point of discharge, based on their clinical judgement of admission necessity.

A patient satisfaction survey was designed and disseminated to patients in the ED to collect feedback; surveys were completed voluntarily and anonymously before discharge. The survey comprised six closed questions with yes/no responses (covering dignity, privacy, communication, and discharge planning) and free‐text space for additional comments (Table 4). AFS clinicians provided paper copies of the survey to patients immediately after completing the ED review. All patients reviewed by the AFS were eligible to participate. However, surveys were only offered to patients whom clinicians judged able to provide informed feedback. Patients with severe cognitive impairment (e.g., advanced dementia, delirium) without a family member present to assist were not routinely offered the survey. Family members or carers could assist with completion if the patient consented and was present.

### 3.6 Outcome Measures

#### 3.6.1 Primary Outcomes Were

- hospital length of stay: Total duration from ED arrival time to hospital discharge (for admitted patients) or ED discharge time (for patients discharged directly from ED). This encompassed the entire index presentation only—time spent in the ED, short‐stay unit, and inpatient wards until discharge. LOS was calculated in hours and analysed both as continuous and log‐transformed variables. Reattendances were not included in LOS calculations.
- hospital admission rates (including admission avoidance): Admission to an inpatient ward (including short‐stay units) from the ED. Patients discharged directly home, to a care home, or to community services (e.g., Hospital at Home) were classified as not admitted.

#### 3.6.2 Secondary Outcomes Included

- 30‐day reattendance: Any unplanned return to the ED within 30 days of the index presentation discharge date. This included reattendances for any cause, not limited to the same presenting complaint. Planned follow‐up appointments, elective admissions, and primary care contacts were excluded.
- inpatient mortality: Death occurring during the index hospital admission (for admitted patients) or within ED attendance (for patients discharged directly from ED). Mortality during any subsequent readmissions was not included in this outcome.
- estimated cost avoidance based on the number of admissions avoided: Number of patients discharged following AFS review who, in the clinical judgement of the AFS clinician, would have been admitted to hospital had the AFS intervention not been available. This judgement was documented prospectively at the point of discharge.
- patient satisfaction, captured through a short, structured survey on communication, involvement, and continuity of care.

We used the outcome measures for CGA reported in previous literature to evaluate this service; however, it is worth noting that the intervention provided by AFS involves initiating CGA, with or without community follow‐up, to complement CGA further. This was not designed to test efficacy, but to explore feasibility and detect potential signals of benefit.

### 3.7 Ethics Approval and Consent to Participate

This project was reviewed by the Clinical Governance Department and Research Support Manager at Oxford University Hospitals NHS Foundation Trust (OUH). It was classified as a Service Evaluation/Service Improvement project in accordance with UK Health Research Authority (HRA) guidance and therefore did not require Research Ethics Committee review.

The activity was registered and approved as a local clinical audit (Audit No. 9409) within the OUH Clinical Audit Programme. Departmental approval was obtained, and all procedures complied with OUH clinical governance requirements.

As this evaluation used anonymised routinely collected service data, individual consent for data use was not required. Patient feedback was collected voluntarily and anonymously as part of routine service monitoring, and completion of the survey was taken as implied consent.

## 4 Data Analysis

Descriptive statistics were used to summarise baseline characteristics, outcomes, and subgroup comparisons. Hospital length of stay (LOS) was the primary continuous outcome. LOS data were positively skewed; analyses were conducted using both untransformed and log‐transformed values. Between‐group comparisons of LOS (frailty intervention vs. usual care) were examined using analysis of variance. Where appropriate, analysis of covariance was used to adjust for age and baseline CFS scores. Associations between age, CFS, and LOS were further explored using Pearson's and partial correlations. Odds ratios (ORs) with 95% confidence intervals (CIs) were calculated to estimate the association between frailty intervention and outcomes including hospital admission and 30‐day reattendance. Further subgroup analysis can be found in the supporting material (Appendix 4). A two‐sided significance threshold of *p* < 0.05 was applied throughout. Missing data were assessed for randomness using Little's MCAR test. The test was not significant (*p* = 0.42), indicating that data were missing completely at random. Consequently, listwise deletion was applied, resulting in the exclusion of cases with missing values from the analyses. All analyses were conducted using SPSS version 29 (IBM, Armonk, NY). A preliminary cost‐offset estimate was calculated by the number of admissions avoided per clinician day. Patient satisfaction was summarised as the percentage of respondents providing positive responses across survey domains.

## 5 Results

### 5.1 Baseline Characteristics

During the evaluation period, 5717 patients aged ≥ 65 with a CFS ≥ 6 presented to the ED. Of these, 464 (8.1%) were reviewed by the AFS and 5253 (91.9%) received standard ED care. Patients seen by AFS were older (mean age 85.9 vs. 81.0 years), more likely to be female (64.2% vs. 55.3%), and had higher rates of moderate frailty (CFS 6–7). The majority of patients in both groups had low NEWS2 scores on arrival, but AFS patients were more frequently discharged home from the ED (34.7% vs. 24.5%) (Table 2).

**Table 2:** Comparing ED patients seen by AFS and not seen by AFS.

		Patients presented to ED not seen by AFS *n* = 5253	Patients presented to ED seen by AFS *n* = 464
Age (*years*)	*Mean*	81.0	85.9
	SD	12.3	7.7
Gender	*Female*	2903 (55.3%)	298 (64.2%)
	*Male*	2350 (44.7%)	166 (35.8%)
Ethnicity	*White*	4127 (78.6%)	385 (83.0%)
	*Non‐white*	281 (5.3%)	21 (4.5%)
	*Not known*	845 (16.1%)	58 (12.5%)
Discharge Destination	*A&E*	35 (0.7%)	2 (0.4%)
	*Home*	1288 (24.5%)	161 (34.7%)
	*Nursing Home*	54 (1.0%)	7 (1.5%)
	*Residential Home*	13 (0.2%)	3 (0.6%)
	*Wards*	1157 (22.0%)	80 (17.2%)
	*ED* →*Short stay*	2526 (48.1%)	206 (44.4%)
	*ED* → *SDEC*	3 (0.1%)	1 (0.2%)
	*All other categories*	47 (0.9%)	4 (0.9%)
Clinical Frailty Score	*6*	3289 (62.6%)	326 (70.3%)
	*7*	1540 (29.3%)	127 (27.3%)
	*8*	307 (5.8%)	10 (2.2%)
	*9*	117 (2.2%)	1 (0.2%)
Track and Trigger Score (NEWS2)	*Low* [0–4]	4519 (86.0%)	446 (96.1%)
	*Medium* [5–6]	253 (4.8%)	7 (1.5%)
	*High* [≥ 7]	481 (9.2%)	6 (1.3%)
	*Not known*	0 (0%)	5 (1.1%)

*Note:* SDEC: Same Day Emergency Care; NEWS2: National Early Warning Score 2.

### 5.2 The Effect of ED Frailty Intervention Provided by the AFS on the Length of Hospital Stay (LOS)

AFS intervention was associated with a shorter LOS overall (49.1 ± 48.5 h vs 106.4 ± 95.5 h, *p* < 0.001) (Appendix 2 Supplementary Figure 1), driven primarily by admitted patients. Among patients discharged from the ED, LOS was not significantly different. Sensitivity analyses using log‐transformed LOS confirmed these trends (Appendix 3 Supplementary Figure 2). However, this likely reflects patient selection, as the service often reviews individuals who are more suitable for discharge (e.g., falls without fractures). Therefore, while a trend towards reduced LOS was observed, these findings should be interpreted with caution and considered exploratory rather than causal.

### 5.3 Associations Between AFS and Subsequent Hospital Admission/Reattendance

Patients reviewed by AFS were less likely to be admitted (OR 0.14, 95% CI 0.087–0.231) (Table 3), although this likely reflects patient selection. No significant associations were observed for 30‐day reattendance (OR 0.84, 95% CI 0.51–1.36) or Hospital at Home referral (OR 1.38, 95% CI 0.85–2.25). Odds ratio analyses were conducted on subsets of patients with complete data for the relevant outcome and disposition categories; therefore, denominators differ from the overall cohort.

**Table 3:** Odds ratios (ORS) and 95% confidence intervals (CIs) to assess associations between AFS and hospital admission/reattendance within 30 days.

Table 3A	Admission	Discharge	*Odds*
Patients who *received* ED frailty intervention	*n* = 23	*n* = 61	0.377
Patients who *did not receive* any ED frailty intervention	*n* = 1461	*n* = 549	2.661
			OR = 0.142* [0.087, 0.231]
Table 3B	Reattendance	No reattendance	*Odds*
Patients who *received* ED frailty intervention	*n* = 5	*n* = 56	0.089
Patients who *did not receive* any ED frailty intervention	*n* = 53	*n* = 496	0.107
			OR = 0.836^n.s.^ [0.512, 1.363]
Table 3C	Reattendance	No reattendance	*Odds*
Patients who *were referred* to the Hospital at Home (H@H) service	*n* = 3	*n* = 45	0.067
Patients who *were not referred* to any H@H service	*n* = 65	*n* = 1347	0.048
			OR = 1.381^n.s.^ [0.847, 2.254]

*Note:* * = *p* < 0.05, *n.s*. = not significant.

### 5.4 In‐Hospital Mortality

Inpatient mortality during the index admission was recorded for 0% of AFS‐reviewed patients between September 2023 and March 2024. Given the absence of events during index admissions, no comparative analyses were conducted.

### 5.5 Preliminary Cost‐Offset Estimate of the Service

Preliminary estimates of admission avoidance were calculated by dividing the total number of admissions avoided by the number of clinician shifts, resulting in an average of two admissions avoided per shift. Using a local cost estimate of £500 per patient bed, this suggests that the potential savings could offset the costs of clinician staffing.

While these findings suggest that the AFS may be economically beneficial, they represent a simple cost‐offset calculation rather than a formal cost‐effectiveness analysis. A comprehensive economic evaluation, encompassing all service costs, patient outcomes, and sensitivity analyses, would be necessary to determine the true cost‐effectiveness of the service.

### 5.6 Patient Satisfaction With the Service

A total of 24 responses were received from patients seen by the AFS in the ED. Table 4 summarises the patient responses to the survey questions. Most patients expressed satisfaction with the service; however, it was noted that clearly explaining the next steps in their care can be challenging, as the plan may change based on the results of investigations. Table 5 includes some comments from patients and their families.

**Table 4:** Patient response to the survey question.

Survey questions	Response of yes percentages
Did you feel you were treated with respect and dignity?	23/24 (95.8%)
Did we assess you in a comfortable and private environment?	24/24 (100%)
Were you offered food and/or drink?	23/24 (95.8%)
Did we explain the tests, treatments and diagnoses that have happened today?	22/24 (91.7%)
Did we answer all your questions?	24/24 (100%)
Did we explain enough about your discharge and what happens next?	21/24 (87.5%)

**Table 5:** Illustrative comments from patients and families regarding their experience with the acute frailty service in the emergency department.

“The doctor and nurse were very friendly and professional and treated Mum with respect, kindness and dignity.” “Thank you for your support and help. It's been a daunting time for us all navigating out way through all the processes.” “I feel very comfortable. The doctor explained she was going to stop my medication that cause me to be dizzy.” “I didn't like being moved into a dungeon like room which is dark, stuffy and has no windows. I think daylight is important and don't think people should be deprived of it.” “Wasn't offered more water. Don't feel that testes were explained well/if at all. Answered my questions eventually ‐ think doctor needed to speak to daughter first.” “We were looked after very well; all the nurses and doctor were very helpful and understanding.” “I was made to feel welcome and excellent. I was looked after by the nursing staff.” “I am at a difficult time as my husband is very ill. You have been my understanding. I think a little more information about what is happening in between being seen by the doctor and going home (would be good).”

## 6 Discussion

Of 5717 eligible patients (≥ 65 years, CFS ≥ 6), only 464 (8.1%) were reviewed by the AFS. This low review rate reflects operational constraints rather than clinical selection alone: the AFS operated Monday–Friday, 9 a.m.–5 p.m. only, meaning weekend and nighttime attendees (approximately 40% of presentations) were ineligible for review; the small team of one senior nurse and one geriatric registrar had limited daily review capacity, prioritising patients most suitable for admission avoidance; reliance on ED staff referrals alongside proactive screening may have resulted in inconsistent case identification during early implementation; and the service focused on stable patients with admission avoidance potential, meaning higher‐acuity patients were less likely to be prioritised. The AFS population, therefore, represents a selected subgroup of frail older adults—those presenting during weekdays during daytime with stable presentations perceived as suitable for community discharge.

This service evaluation suggests that an ED‐based frailty service can support admission avoidance without compromising patient flow and is well‐received by patients. Although this evaluation was conducted at a single UK site, the findings align with international evidence supporting early geriatric assessment in acute care, such as NHS CQUIN priorities, and resonate with global policy frameworks, including the WHO's calls for integrated care for older adults (WHO ICOPE) [22] and European initiatives on frailty management (ADVANTAGE Joint Action) [23]. The model demonstrated how integrating frailty expertise into emergency pathways can enhance patient‐centred outcomes and system efficiency. Although conducted in a single UK site, this evaluation offers a transferable model for embedding frailty services in EDs internationally, particularly in health systems facing rising demand from older adults. Patients reviewed by the service had a shorter LOS than other frail ED patients; however, this association is likely influenced by selection bias, as the team frequently targeted patients who were more likely to be safely discharged. As such, LOS findings should be considered exploratory rather than causal. Taken together, these findings demonstrate the feasibility and early outcomes of embedding a frailty‐focused pathway at the ED front door.

Previous studies reported positive benefits of CGA on LOS [12]. However, a recent systematic review found no effect of CGA on hospital length of stay [15]. Determining whether this relationship is causal or merely associative is challenging, as the service selects eligible older adults with frailty based on the eligibility criteria mentioned in the introduction. When taking into account weekdays and weekends, the difference in LOS became insignificant. It is uncertain whether other factors, such as time and date of arrival, which are associated with better staffing or availability of transport, may contribute to the result. In addition, for those patients who were not admitted to the hospital, there was no significant difference in LOS between those who received AFS input and those who did not. This suggests that the provision of an extra clinician assessment did not prolong the LOS of the discharged patients and is unlikely to contribute to reduced ED flow or workload burden. The result also suggests that patients seen by AFS might be less likely to be admitted than patients not seen by AFS, which supports a previous systematic review [24].

As part of CGA initiation, we begin conversations around advance care planning (ACP), including discussions on Do Not Attempt Cardiopulmonary Resuscitation and strategies to avoid unnecessary admissions for older adults with severe frailty. A systematic review by Sakamoto et al. [25] found that ACP can lead to fewer ED visits and ambulance calls among residents in care homes. Additionally, Haynesworth et al. [26] reported that CGA reduced the risks of both admission and reattendance within 30 days, aligning with other studies that highlight the benefits of CGA on reattendance rates [27, 28, 29].

We anticipate that reattendance to the ED (for all causes) would likely decrease when comparing patients under the intervention to other frail individuals in the ED, as ED clinicians are less likely to initiate such conversations. Our findings indicate that conducting a front‐door frailty review and subsequent referral to H@H did not lower the ED 30‐day reattendance rate. This aligns with a previous study by Heeren et al. [30], which found no reduction in unplanned reattendances following a geriatric assessment.

It is important to note that patients referred to H@H were often perceived as having unresolved acute medical needs. Therefore, these patients may have had a higher likelihood of reattendance even before the referral. Consequently, having a similar revisit rate as those not referred to H@H may not necessarily indicate no impact on revisits. Future studies may benefit from a before‐and‐after design with a longer follow‐up period to assess the effect of front‐door CGA on preventing hospital admissions.

Overall, this evaluation demonstrates the feasibility and acceptability of introducing a dedicated frailty service in a busy ED setting. The notable benefits observed were safe admission avoidance, preserved ED flow, and positive patient experience. While there were signals of reduced LOS, these must be interpreted with caution due to selection bias and uncontrolled confounding. Larger, prospective studies are required to establish the true impact of such services on hospital outcomes.

In summary, this study contributes real‐world evidence on implementing frailty‐focused pathways in emergency settings. The acute frailty service provided measurable benefits for older adults while maintaining ED efficiency. Although limited by selection bias and the pragmatic design, these findings underline the importance of integrating specialist geriatric input at the point of presentation. Future research should include prospective, multi‐centre evaluations examining long‐term patient outcomes, cost‐effectiveness, and scalability of similar models across diverse healthcare systems.

## 7 Limitations of This Service Evaluation

This service evaluation has several limitations. Although analyses adjusted for age and frailty score, the intervention inherently involved the selection of patients perceived as more likely to benefit or be safely discharged. For example, individuals with falls but no fractures were more frequently reviewed, whereas those with hip fractures or higher acuity were not. This introduces selection bias and limits the interpretation of outcomes such as LOS, which may therefore reflect case mix as much as service impact. Selection bias is inherent in early service evaluations. However, feasibility studies are essential precursors to randomised trials and provide pragmatic insights for service design and scale‐up. Another limitation of our data collection was the reliance on the researcher's judgment to determine whether ‘equivalent' assessments occurred in the usual care group. Despite using predefined criteria and dual review, some assessments may have been missed or misclassified due to inconsistent documentation in clinical notes.

Several methodological limitations should be noted regarding outcome measurement. Reattendance data, while extracted by informatics staff blinded to intervention status, included any‐cause emergency returns and did not distinguish between related and unrelated presentations. Admission avoidance was based on unvalidated clinician judgement at the point of discharge, which may have been influenced by awareness of service goals or availability of community pathways. No inter‐rater reliability assessment was conducted for these judgements. Patient satisfaction data were collected from a small, self‐selecting sample and may not be representative of all patients seen by the service.

Other unmeasured factors, such as the day and time of arrival (weekday vs. weekend) and access to transportation or community services, may have influenced LOS and admission decisions. Third, the evaluation period was short, and follow‐up was limited to 30 days; therefore, longer‐term outcomes, such as sustained admission avoidance or functional recovery, were not captured. Finally, and importantly, we did not examine the quality or comprehensiveness of care delivered. While we recorded whether patients received therapy review or medication review, we did not assess whether those seen by AFS received a better level of CGA than those in usual care—for example, whether CGA domains were more completely addressed, whether advance care planning discussions were initiated, or whether patients received more referrals to outpatient frailty services, falls prevention programmes, or community geriatric follow‐up. It is therefore possible that some of the observed differences between groups reflect not simply the presence of AFS input, but the intensity and completeness of the assessment delivered. Future evaluations should incorporate measures of CGA quality and downstream frailty interventions to better understand the mechanisms by which front‐door frailty services may improve outcomes. These limitations are common to real‐world service evaluations and reflect the pragmatic nature of implementing new frailty pathways in busy acute settings.

## 8 Conclusion

This service evaluation explored the implementation of an acute frailty service in a large urban ED. The evaluation indicates that the service was feasible to deliver, did not delay ED flow, supported safe admission avoidance, and was viewed positively by patients and families. While patients reviewed by the service had shorter hospital LOS compared with other frail ED patients, this finding is likely influenced by selection bias and should be interpreted cautiously. Overall, the notable benefits of the service lie in safe admission avoidance, patient satisfaction, and demonstrating that frailty‐focused pathways can be embedded in a busy ED environment. The observed signals around LOS highlight an area for future prospective studies with larger samples and stronger control for confounding. Further evaluation is warranted to establish the long‐term outcomes and cost‐effectiveness of acute frailty services in emergency care.

## Funding

The authors have nothing to report.

## Ethics Statement

This work was registered with the hospital's clinical governance team as a service evaluation. According to national guidance, formal research ethics approval was not required.

## Conflicts of Interest

The authors declare no conflicts of interest.

## Supporting information

Appendix 1.

Appendix 2.

Appendix 3.

Appendix 4.

## Data Availability

The data that support the findings of this study are available on request from the corresponding author. The data are not publicly available due to privacy or ethical restrictions.
