# Supplementary material for: Implementing an Acute Frailty Service in the Emergency Department: A Mixed‐Methods Service Evaluation of Feasibility, Patient Outcomes and Experience
Source: J Eval Clin Pract. 2026 Mar 30;32(3):e70432. doi: 10.1111/jep.70432 (PMC13035255; doi:10.1111/jep.70432)
Supplement: Supplementary file 2 — Appendix 2. [file JEP-32-0-s004.docx]

**Appendix 2** Supplementary *Fig.* 1


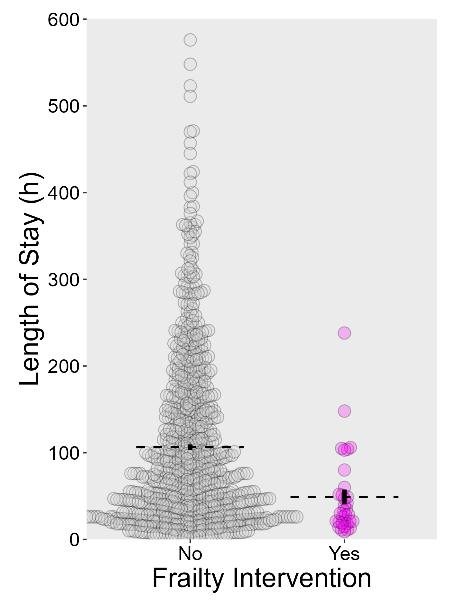

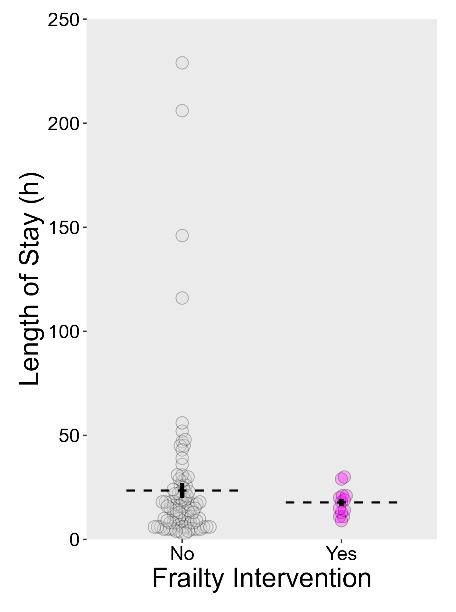

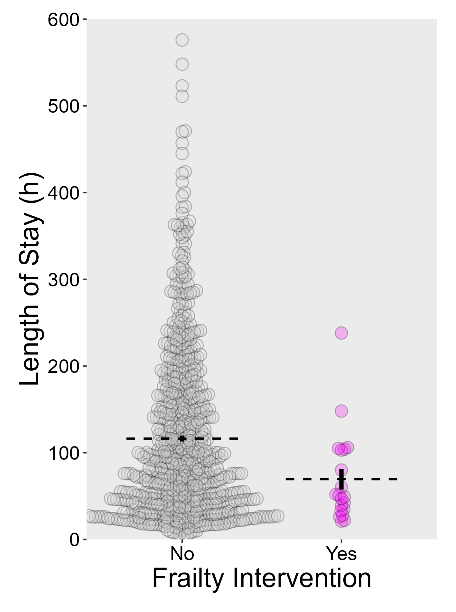


Supplementary *Fig.* 1. Mean length of stay (LOS) for frail ED patients (≥65 years, CFS ≥6)

*Left* panel: All patients (AFS vs non-AFS)

*Middle* panel: Subgroup – discharged from ED

*Right* panel: Subgroup – admitted from ED

Patients reviewed by the AFS had shorter LOS overall compared with non-AFS patients (49.1h vs 106.4h, p<0.001). Subgroup analysis showed no significant difference for discharged patients (p=0.56), but a significant reduction for admitted patients (96.2h vs 123.8h, p=0.03).

Subgroup analyses revealed differential effects by patient disposition. For patients discharged directly from the ED (N = 13 intervention vs N = 95 non-intervention), the reduction in LOS was not statistically significant (F(1,106) = 0.340, p = 0.561, partial η² = 0.003), although a trend toward shorter LOS was observed. Among patients admitted to the hospital from the ED (N= 20 intervention vs N = 797 non-intervention), AFS intervention was associated with a significant decrease in LOS (96.2 h vs 123.8 h; F(1,815) = 4.760, p = 0.029, partial η² = 0.006). This effect remained significant after adjusting for age and CFS in an ANCOVA (F(1,813) = 4.838, p = 0.028, partial η² = 0.006), indicating that baseline patient characteristics did not explain the reduction in LOS.
