# Supplementary material for: Implementing an Acute Frailty Service in the Emergency Department: A Mixed‐Methods Service Evaluation of Feasibility, Patient Outcomes and Experience
Source: J Eval Clin Pract. 2026 Mar 30;32(3):e70432. doi: 10.1111/jep.70432 (PMC13035255; doi:10.1111/jep.70432)
Supplement: Supplementary file 3 — Appendix 3. [file JEP-32-0-s002.docx]

**Appendix 3:** Supplementary *Fig.* 2

**
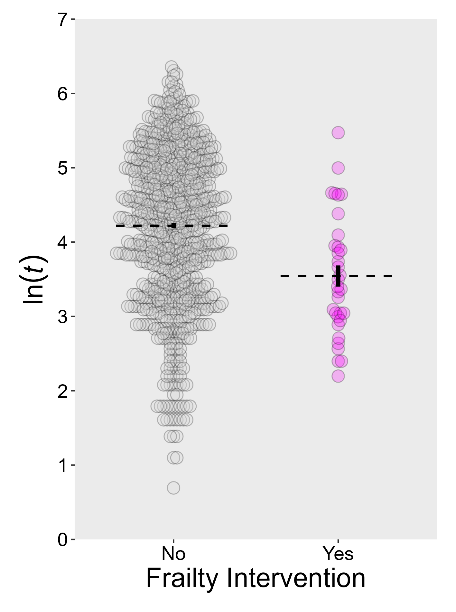

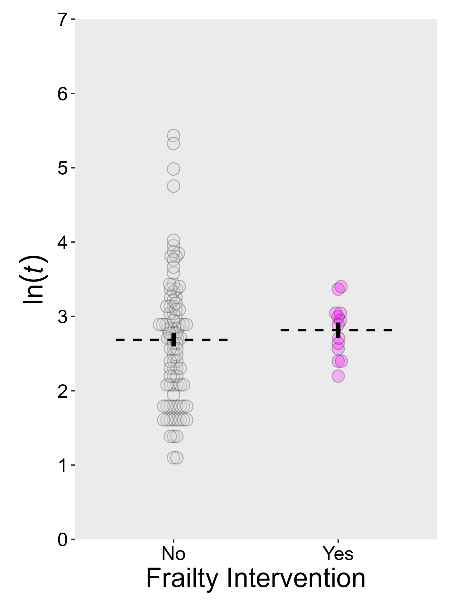

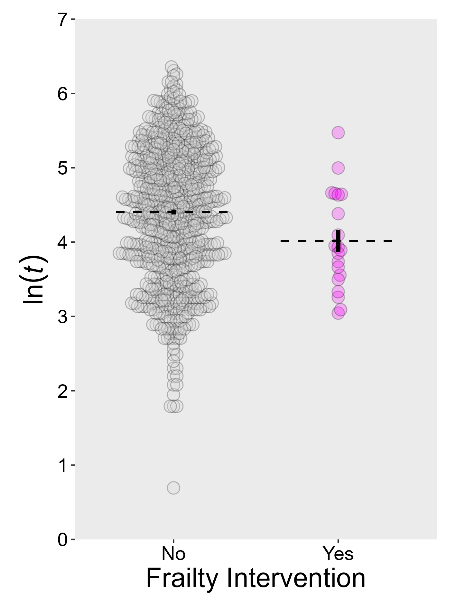
**

Supplementary *Fig.* 2. The Effect of AFS on Log-transformed Length of Hospital Stay

LOS data were positively skewed and therefore log-transformed [ln(t)] to meet the assumptions of normality and homogeneity of variance for ANOVA.

- **Left panel:** Means ± standard errors of the mean (SEMs) of ln(LOS) for all patients not receiving frailty intervention (***N*** = 892; grey) versus patients receiving ED frailty intervention (***N*** = 33; magenta), **p < 0.001**.
- **Middle panel:** Patients discharged directly from the ED (***N*** = 95 non-intervention; ***N*** = 13 intervention). No significant effect of AFS intervention was observed (**p = 0.601**).
- **Right panel:** Patients admitted to the hospital from the ED (***N*** = 797 non-intervention; ***N*** = 20 intervention). The effect of AFS intervention approached significance (**p = 0.056**).

Separate one-way ANOVAs were conducted for each panel. These results confirm the trend toward reduced LOS with AFS intervention, particularly among admitted patients.
