# Supplementary material for: Implementing an Acute Frailty Service in the Emergency Department: A Mixed‐Methods Service Evaluation of Feasibility, Patient Outcomes and Experience
Source: J Eval Clin Pract. 2026 Mar 30;32(3):e70432. doi: 10.1111/jep.70432 (PMC13035255; doi:10.1111/jep.70432)
Supplement: Supplementary file 4 — Appendix 4. [file JEP-32-0-s003.docx]

**Appendix 4**

### Subgroup Analysis I: The Effect of AFS on Patients presenting with falls

To explore if there was any effect of ED frailty intervention (AFS) specifically for patients coming with falls (selected as a controlled case comparison; Age mean 86.1± 7.3 VS 86.9± 6.0, p= 0.433; Pearson Chi-Square for CFS *p* = 0.51), excluding severely unwell patients such as hip fractures, severe trauma, palliative patients and patients admitted into intensive care units), an ANCOVA was conducted in patients presenting with falls who received frailty intervention versus those who did not receive any frailty intervention (*n* = 18 *versus* *n* = 61). There remains a significant effect of frailty intervention on LOS in the ANCOVA with patients’ age and CFS as covariates; like the main analyses, the mean LOS in patients presenting with falls who received ED frailty intervention, *mean* = 39.8 h and *SD* = 32.2 h, was less when compared to the mean LOS in patients presenting with falls who did not receive similar services, *mean* = 97.0 h and *SD* = 109.0 h [*F*(1,75) = 4.687, *p* = 0.034, partial *η*^2^ = 0.059]. However, the difference became insignificant when the factor of arrival time taken into account (weekdays). Table 3 illustrates the demographics and outcomes of a further subgroup of patients who had a fall with no abnormality detected in the ED.

These results suggest a trend towards shorter LOS in patients with falls who were reviewed by the service. However, this may be explained by patient selection and temporal factors (e.g., weekday vs weekend arrivals), so findings should be considered exploratory. Overall, AFS intervention appears to offer meaningful efficiency benefits in this clinically stable subgroup presenting with falls without adversely affecting short-term readmission or discharge outcomes.

**Supplementary Table 1**

**The Demographics and Outcomes of Patients Presenting with a Fall with No Abnormality Detected**

|  |  | **No AFS**  (*n* = 15) | **AFS**  (*n* = 17) |
| --- | --- | --- | --- |
| Gender | *Female* | 11 (73.3%) | 14 (82.4%) |
|  | *Male* | 4 (26.7%) | 3 (17.6%) |
| Baseline Care | *No care* | 4 (26.7%) | 2 (11.8%) |
|  | *Care home* | 5 (33.3%) | 7 (41.2%) |
|  | *POC (unknown frequency)* | 1 (6.7%) | 0 (0%) |
|  | *BD POC* | 0 (0%) | 4 (23.5%) |
|  | *TDS POC* | 2 (13.3%) | 2 (11.8%) |
|  | *QDS POC* | 1 (6.7%) | 1 (5.9%) |
|  | *OD POC* | 0 (0%) | 1 (5.9%) |
|  | *No POC* | 2 (13.3%) | 0 (0%) |
| Discharge Destination | *No care* | 3 (20.0%) | 1 (5.9%) |
|  | *Care home* | 5 (33.3%) | 7 (41.2%) |
|  | *POC (unknown frequency)* | 1 (6.7%) | 1 (5.9%) |
|  | *BD POC* | 0 (0%) | 2 (11.8%) |
|  | *TDS POC* | 1 (6.7%) | 2 (11.8%) |
|  | *QDS POC* | 2 (13.3%) | 1 (5.9%) |
|  | *OD POC* | 0 (0%) | 1 (5.9%) |
|  | *No POC* | 1 (6.7%) | 0 (0%) |
|  | *Hub bed* | 1 (6.7%) | 1 (5.9%) |
|  | *Community Hospital* | 1 (6.7%) | 1 (5.9%) |
| Age (*years*) | *Mean* | 83.3 | 84.8 |
|  | *SD* | 8.2 | 7.2 |
| National Early Warning Scale | *0* | 1 (6.2%) | 0 (0%) |
|  | *1* | 11 (68.8%) | 14 (82.3%) |
|  | *2* | 4 (25%) | 3 (17.6%) |
| Clinical Frailty Score | *6* | 7 (46.7%) | 10 (58.8%) |
|  | *7* | 7 (46.7%) | 5 (29.4%) |
|  | *8* | 1 (6.7%) | 2 (11.8%) |
| Length of Stay (hour*s*) | *Mean* | 57.5 | 24.6 |
|  | *SD* | 107.0 | 30.6 |
| Admission | *No* | 12 (80.0%) | 14 (82.4%) |
|  | *Yes* | 3 (20.0%) | 3 (17.6%) |
| Reattendance within 1 month | *No* | 12 (80.0%) | 12 (70.6%) |
|  | *Yes* | 3 (20.0%) | 5 (29.4%) |

**Note:** Patients included all arrived in ED on weekdays (Monday to Friday)

### Subgroup Analysis II: The Effects of Services Provided by Frailty Intervention Team (FIT) *versus* AFS in Discharged Patients who bedded under ED

To explore the effects of the services provided by both the frailty intervention team (FIT and AFS), a between-subjects ANOVA was conducted on LOS in *discharged* patients, who were divided into the following categories: patients without FIT or AFS (*n* = 61 *FIT*−*AFS*− patients); those who received either one of the two services (*n* = 18 *FIT*+*AFS*− and *n* = 2 *FIT*−*AFS*+ patients); and those who received both services (*n* = 16 *FIT*+*AFS*+ patients). The main effect of Group fell short of significance at the conventional *α* = 0.05 level [*F*(3,93) = 2.457, *p* = 0.068, partial *η*^2^ = 0.073]; however, further analyses revealed a difference between *FIT*−*AFS*− and *FIT*+*AFS*− patients [mean difference = 18.3 h, *post hoc* Bonferroni *p* = 0.053 (*grey* boxes in Supplementary **Table 2**)]. All other comparisons were not significant (*p* > 0.6), suggesting that the addition of AFS did not prolong LOS in discharged patients who received FIT.

Lastly, to assess the strength of associations between both frailty services (including FIT and AFS) and subsequent hospital reattendance in *discharged* patients only, *reattendance*/*no-reattendance*ORs and corresponding CIs were calculated in Supplementary **Table 3**. These ORs varied between discharged patients that received either one of the two frailty services (either ***FIT*−*AFS*+** or ***FIT*+*AFS*−**) *versus* those who received both services (***FIT*+*AFS*+**). In ***FIT*+*AFS*−** patients, the FIT service alone was associated with a significant increase in hospital reattendance with an OR of 1.97 and 95% CI of [1.21, 3.21]. By contrast, in ***FIT*+*AFS*+** patients the combination of FIT and AFS services was associated with a non-significant *reduction* in hospital reattendance with an OR of 0.80 and 95% CI of [0.49, 1.31]. Thus, the clinical outcome of ED frailty intervention in certain patient subgroups could potentially be optimised by combining both FIT and AFS.

These findings indicate that, in discharged patients, LOS is largely unaffected by the addition of AFS to FIT services, while reattendance outcomes may be optimised when both services are provided together. The combination of FIT and AFS appears to offer the potential to improve clinical outcomes compared with either service alone, highlighting the value of integrated frailty interventions in selected ED patient subgroups.

**Supplementary Table 2**

**Means and Standard Deviations (SDs) of Length of Stay (LOS) in Discharged Patients With and Without Frailty Intervention Team (*FIT*) or AFS**

|  | ***FIT*−** | ***FIT*+** |
| --- | --- | --- |
| ***AFS*−** | *mean* = 15.3 h, *SD* = 16.9 h  *n* = 61 | *mean* = 33.6 h, *SD* = 50.2 h  *n* = 18 |
| ***AFS*+** | *mean* = 11.0 h, *SD* = 0 h  *n* = 2 | *mean* = 19.6 h, *SD* = 6.1 h  *n* = 16 |

**Supplementary Table 3**

**Odds Ratios (ORs) and 95% Confidence Intervals (CIs) to Assess Associations Between FIT/AFS and Hospital Reattendance in Discharged Patients**

| Patients | Reattendance | No reattendance | *Odds* | *OR* [95% CI] |
| --- | --- | --- | --- | --- |
| Control ***F*−*A*−** | *n* = 30 | *n* = 450 | 0.067 |  |
| ***F*−*A*+** | *n* = 7 | *n* = 71 | 0.099 | 1.479 [0.91, 2.41] *^n.s.^* |
| ***F*+*A*−** | *n* = 24 | *n* = 183 | 0.131 | 1.967 [1.21, 3.21] ***** |
| ***F*+*A*+** | *n* = 36 | *n* = 674 | 0.053 | 0.801 [0.49, 1.31] *^n.s.^* |
